# Mr. Atkinson, on Hernia

**Published:** 1807-03

**Authors:** James Atkinson

**Affiliations:** York


Gentlemen,

A Young man came recommended to me from the Country:* a spare, dry, parsimonious object, labouring under the usual symptoms and condition of Hernia.

He had endured the pains of this horrid complaint above three days; and was consigned to me in despair, by a very respectable surgeon, who had failed in relieving him.

The patient, after long struggling with a sheep, and in the act of throwing it upon his shoulders, suddenly felt the descent of a rupture. He knew well his complaint, from having once suffered it.

The scrotum and spermatic process were hard and tense up to the ring; but not very large or very full. A single turn of the intestine appeared.

The pain was incessant, though by no means so acute as it had been. He was constantly sick, and frequently retching; nothing being admitted since the accident to stay upon his stomach. He had considerable pain over the abdomen, with a wistful and miserable look. In short, obstipated and restless, he laboured under the general symptoms of strangulated Hernia. He came as a forlorn hope to me, and as such I considered him.

Continued affusion of cold water, † and all the attempts or means of reducing it used by a friend ‡ and myself, were unavailing; nor did circumstances apparently countenance the operation. At least before this anceps remedium were allowed, I determined to see the effect which would arise from puncturing the intestine.

This was performed with a male trocar, at the most expedient side of the scrotum. There issued immediately bloody serum; which being permitted to flow its own time, produced in quantity near half a pint.

The consequence.—A collapse of the intestine and scrotum promptly took place, and in a few minutes, by gentle use of the fingers, the intestine being returned, it gave instant ease.

Am

---

* November 17th.

† From a washing-tub, perhaps unfortunately not in Mr. Little's way.

‡ Mr. Saunders.

Am I sanguine, or premature, in considering this fact of some consequence in practice?

When the scrotum and intestine were turgid, proper pressure could not be used to advantage. On future occasions, neither pressure nor taxis may be required, possibly the contents may return *suâ sponte*. In these respects some benefit may be derived, providing that the bad consequences do not more than counterpoise those which are good.

Solitary as the case may be, on evidence, thus far stands the result.

I wave a minute description of the progress, as tedious and unnecessary. Suffice it to observe, that nearly three weeks elapsed, before the symptoms of irritability of the stomach subsided; or before the functions of the intestines were duly restored. And these objects were happily effected, only by the most gradual and guarded procedure.

The patient suffered most after the operation, from a painful inflation of the intestines and abdomen, and from a very palpable accumulation of fluid.

The murmur of the intestines, and there tumbling over en culbut, was altogether new and surprising. A person less grave than myself, might have stiled it ridiculous.— Indeed it was quite amasing to the old nurse in attendance; and this inflation, &c. is not ill expressed by the bombast and barbarous term Borborygmus.

Frequent injections, aperient medicines, &c. &c. were long tried in vain. Many days passed before he had an evacuation per anum, or could retain or relish food. Saline purgatives invariably returned. Castor oil and calomel answered better.

No air, or collection or flow of serum continued from the scrotum.

This case proved uncommonly troublesome, as the patient was of that species of being, whose indifference forms a character so prevalent in the country; a character in whom the senses, except in matters of money, scarce appear to exist.

The relaxation of confinement, of bed, &c. produced a Hernia on the other side. The scrotum and spermatic process soon subsided after the puncture, and almost entirely recovered the effects of handling and of contusion. The original Hernia sometimes came partially down; but when the parts could bear it, a double truss relieved the accident, accident, and remedied the inconvenience. He returned to the country,* and speedily recovered.

The history of the case, and the danger being now over, permit a few words, astray from the subject. Never sure in so short a time, was youth more reduced in person. He was about six feet high, and only 17 years old; a terrible long legged type, and absolute cadaveris crates. Near three weeks had he passed, almost without meat or drink. His parched skin upon the abdomen was an exact model of the convolutions of the intestines, clinging to them like ancient drapery. It was precisely the squallid form and figure within an old sarcophagus. He was a spectre, a phantasmagoria, which Callot might have skilfully transformed into an academy figure.

But I should not have ventured thus prematurely to have obtruded this *single* experiment upon the public, unless I had been well aware, how few opportunities the practice of an individual affords, when compared with those of the multitude.

They who consider the above, as an unwarrantable and a rash experiment, probably will not have recourse to it; yet the chance remains, that others may see it in a different light.

I am, &c.

JAMES ATKINSON.

*York, January* 11, 1807.

---

